# On the (in)efficiency of fuzzing network protocols

**DOI:** 10.1007/s12243-024-01058-w

**Published:** 2024-08-13

**Authors:** Seyed Behnam Andarzian, Cristian Daniele, Erik Poll

**Affiliations:** Radboud Universiteit, Nijmegen, Netherlands

**Keywords:** Testing, Fuzzing, Software security, Network protocol fuzzing

## Abstract

Fuzzing is a widely used and effective technique to test software. Unfortunately, certain systems, including network protocols, are more challenging to fuzz than others. An important complication with fuzzing network protocols is that this tends to be a slow process, which is problematic as fuzzing involves many test inputs. This article analyzes the root causes behind the inefficiency of fuzzing network protocols and strategies to avoid them. It extends our earlier work on network protocol fuzzers, which explored some of these strategies, to give a more comprehensive overview of overheads in fuzzing and ways to reduce them.

## Introduction

Fuzzing (a.k.a. fuzz testing) is an effective technique for testing software systems. Popular fuzzers such as AFL++ [23] and LibFuzzer [2] have found thousands of bugs in open-source and commercial software. For example, Google has discovered over 25,000 bugs in their software (e.g., Chrome) and over 36,000 bugs in over 550 open-source projects [4]. Fuzzing involves sending many—tens or hundreds of thousands—(semi)automatically generated inputs to the System-Under-Test (SUT), so the speed of generating and processing many inputs is important.

Unfortunately, not all software can benefit from such fuzzer performance. For instance, network protocol fuzzers struggle to achieve high speeds. Whereas a typical fuzzing campaign with a modern fuzzer like AFL++ [23] on, say, a graphics library will produce thousands of inputs per second [32], a fuzzer like AFLNet [14] for fuzzing network protocols produces only a few dozens of inputs per second. One of the reasons is the overhead of network stacks. To fuzz a network protocol, it is common to modify the code to by-pass the network stack [26]. In addition to this, network protocols are often stateful, and statefulness also influences fuzzer performance [10]: to fuzz a stateful network SUT, a fuzzer is required to send *sequences* of messages (also called traces), instead of a single message. Moreover, context switches between the fuzzer and the SUT can further slow the fuzzing speed here.

This paper focuses on generic techniques that can be implemented in any fuzzer to increase its speed, but we also explore techniques—used in state-of-the-art tools—that require ad hoc modification of the SUT.

In an earlier article [31], we reported results of two different strategies (implemented in a library Desock+ and in a fuzzer called Green-Fuzz) to improve the efficiency of fuzzing network protocols. Desock+ reduces the communication overheads (root cause O1, network stack overhead, in Sect. 3.1). Green-Fuzz reduces the context switches between fuzzer and SUT (root cause O2, context switching, in Sect. 3.2). This article extends the previous paper by comprehensively analyzing the root causes of performance overhead in fuzzing network protocols and strategies to tackle them.

The contribution of the paper is three-fold:

- We analyze the important root causes of overheads in network protocol fuzzing.
- We provide the important strategies to tackle these root causes, including the two presented in the earlier article [31].
- We comprehensively analyze the strategies and their impact, including experimental data to quantify the impact.

Section 2 presents the background. Section 3 delves into the types of overheads encountered in fuzzing network protocols. Section 4 discusses strategies to overcome the communication overheads that slow down the fuzzing network protocols. Section 5 focuses on strategies to reduce the overhead caused by context switching between the fuzzer and the SUT. Section 6 explores strategies to address the initialization and termination overheads. Section 7 analyzes and compares these strategies. In Sect. 8, we review the related work, and in Sect. 9, we discuss future research directions and the limitations of the article. Finally, in Sect. 11, we conclude our article by summarizing our findings and their implications for improving fuzzing performance in network protocol testing.

## Background

In the realm of software security, one of the major challenges is ensuring the robustness and safety of software against malicious inputs. Fuzzing, a dynamic code testing technique, is useful for identifying such vulnerabilities. Performance is very important as fuzzing relies on sending *many* inputs to the SUT.

Time is also critical for integrating fuzzing in the CI/CD^1^ pipelines. As mentioned in [28], the reasonable amount of time that should be spent on fuzzing in the CI/CD pipeline is around 10 min per day, which is very short.

The speed of fuzzing tends to be very poor when fuzzing network protocol implementations. Fuzzing regular command-line software is, on average, 100 times faster than fuzzing network protocol implementations.^2^ This motivated us to do more research and find hurdles in efficiently fuzzing network protocol implementations.

**Fig. 1:**
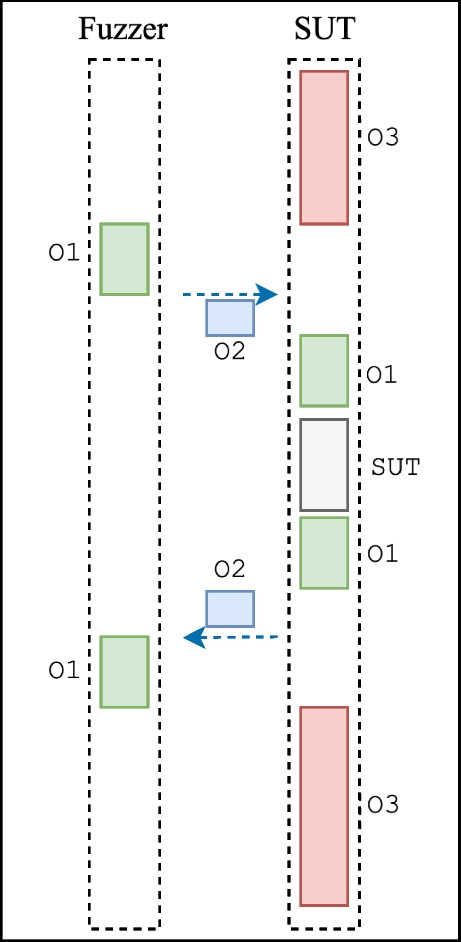
Overheads of fuzzing a network protocol for a trace with one input message. The green color refers to the overhead root cause O1, blue is for the root cause O2, and red is for the root cause O3

## Types of overhead in network protocol fuzzing

In fuzzing network protocols, we identified three kinds of overhead, as shown in Fig. 1:

- **Network stack overhead (O1)** happens when the fuzzer sends the input to the SUT or the SUT sends back a response using the network stack.
- **Context switching overhead (O2)** happens when there is a context switch between the fuzzer and the SUT.
- **SUT initialization and termination overhead (O3)** happens every time the SUT is initialized (i.e., started up) and terminated.

### Network stack overhead (O1)

When testing an application, we can run on the same machine as the fuzzer; the whole network stack is unnecessary. The network stack’s overhead only makes the fuzzing slower and less effective. The network-related system calls also add overhead here. For example, to send a message and receive a response, the fuzzer would need to call the *setsockopt*, *sendto*, and *recvfrom* system-calls.

**Fig. 2:**
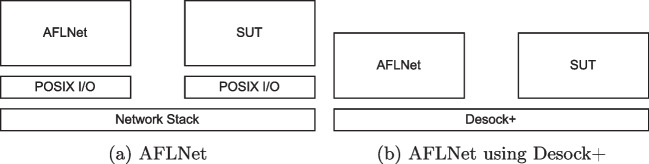
Comparison between plain AFLNet and AFLNet using Desock+

### Context switching overhead (O2)

The context switching overhead in operating systems becomes important when fuzzing network protocols. Fuzzing involves rapidly sending and receiving many inputs and outputs to/from the SUT, which requires frequent context switches between the fuzzer and the SUT. Each context switch entails the operating system saving the state of the currently active process (the fuzzer or the SUT) and loading the state of the other. This can consume significant system resources, especially if there is a high frequency of switches.

Furthermore, the cache invalidation caused by these switches—where previously loaded cache data becomes irrelevant after a context switch—can lead to additional memory reads and hence more overhead. This can significantly affect the efficiency of fuzzing.

### SUT initialization and termination (O3)

In fuzzing network protocols, a new SUT process must be created for each input trace. That process needs to be initialized and involves running constructors and initialization functions. This leads to some *initialization overhead*. After processing each input trace, the SUT must be terminated. The termination process involves the *kill* system call.

This leads to *termination overhead*. This can be expensive as the operating system needs to ensure all resources allocated to the process are properly released.

The cumulative effect of repeatedly initializing and terminating the SUT for each input trace can significantly slow down the fuzzing process. This overhead is not specific to network protocols and is present for fuzzing all types of SUT. Instead of starting a process from scratch for each input, some fuzzers (including AFL) use the *fork* system-call to clone the SUT process to reduce the initialization overhead. That does leave the overhead of the *fork* system-call and does not avoid the overhead of terminating the SUT process.

## Mitigating network stack overhead (O1)

As discussed above, using real network communication to fuzz network protocols introduces overhead. Despite this drawback, it remains a popular choice among many fuzzers, including AFLNet [14], AFLNwe [23], and StateAFL [22]. These fuzzers work by sending inputs and receiving responses through real network sockets and thus suffer the overhead of the—slow—network stack. Strategies to avoid this overhead include (1) using a simulated network stack, (2) using shared-memory between the fuzzer and SUT (which still run as separate processes), and (3) using in-memory communication (which requires fuzzer and SUT to be run as a single process). These three strategies reduce O1 for every input message. We discuss them in more detail below.

### Simulated network stack (Desock+)

Our Desock+ library [31] provides a simulated network socket that works with any fuzzer to avoid network communication overhead. Desock+ does not require emulation or any modification of the source code of the SUT. It is a modified version of the *preeny* library [3], which communicates with the SUT via the standard I/O.

Desock+ can be used by the SUT instead of the standard POSIX library to fuzz more efficiently. The overview of a fuzzer working with Desock+ is shown in Fig. 2. In this case, the fuzzer is a slightly modified version of AFLNet, which sends and receives input messages through standard I/O instead of network sockets. The SUT remains unchanged; the only change is the underlying socket library, which the SUT would load instead of the real socket library.

The difference between *preeny* and Desock+ is that *preeny* can not support specific socket-related system-calls and arguments. Table 1 lists the system calls that Desock+ supports and that allow it to deal with SUTs that

- Contain socket system-calls using blocking or non-blocking network I/O.
- Receive the input messages as data-gram, streams, sequenced, connection-less, and raw.
- Use *connect* and *accept4* system-calls.

The modifications concern the *socket* system-call, which creates the socket file descriptor. More in detail, we added a function named *setup* to modify the socket file descriptor by considering different arguments provided to the socket system-call. Based on the arguments passed to the *socket* system-call, Desock+ uses *fcntl* and *setsockopt* to set different arguments on the socket file descriptor. This way, other socket-related system-calls can use this socket file descriptor without resulting in an error. In *preeny*, these arguments are ignored while creating the socket file descriptor, resulting in an error when other socket-related system-calls try to use different arguments inside the SUT.

Desock+ is only helpful for fuzzing network protocols, whereas *preeny* is also intended to be used for SUT interaction with other services on the system or using a loopback address.^3^ To be able to set different arguments on the socket file descriptor, Desock+ avoids assigning an IP address and port number to the socket file descriptor (setting arguments on a simulated file descriptor with assigned IP and port results in an EINVAL error). However, since *preeny* is meant to be used for many other purposes, this can break its functionality. Therefore, we made Desock+ a separate library.

**Table 1:** Socket-related POSIX system-calls and their arguments supported by Desock+

System-call	Arguments	System-call	Arguments
	*AF_LOCAL*		*SOCK_NONBLOCK*
	*AF_INET*	*connect3()*	*SOCK_CLOEXEC*
	*AF_INET6*		*SOCK_SEQPACKET*
	*SOCK_STREAM*		*SOCK_DGRAM*
*socket()*	*SOCK_DGRAM*		*SOCK_STREAM*
	*SOCK_SEQPACKET*		*SOCK_NONBLOCK*
	*SOCK_RAW*	*dup3()*	*SOCK_CLOEXEC*
	*SOCK_RDM*	*recv()*	*MSG_CMSG_CLOEXEC*
	*SOCK_PACKET*	*recvfrom()*	*SCM_RIGHTS*
	*SOCK_NONBLOCK*	*recvmsg()*	*MSG_DONTWAIT*
*accept4()*	*SOCK_CLOEXEC*		*MSG_ERRQUEUE*
	*SOCK_SEQPACKET*	*send()*	*SOCK_STREAM*
	*SOCK_STREAM*	*sendto()*	*SOCK_SEQPACKET*
*bind()*	*AF_INET*	*sendmsg()*	*MSG_CONFIRM*
	*AF_INET6*		*MSG_DONTWAIT*

### Shared memory

Using shared memory [34] for fuzzing network protocols increases fuzzing network protocols’ performance because communications take place directly through memory rather than actual or simulated network sockets. Unlike Desock+, which relies on files to mimic network communication, shared memory offers a more direct and faster strategy. It eliminates the overheads associated with network stack or file-based communication. So, there is potential to enhance a library further, such as Desock+, by adapting it to use shared memory instead of files.

### In-memory

If we integrate the SUT and fuzzer into a single process—a strategy called in-process fuzzing—then the overhead of passing data between them can still be reduced further. In-process fuzzers can mutate a variable in memory in each fuzzing round. This is called in-memory fuzzing. This differs from shared-memory, where the inputs are sent from the fuzzer process to the SUT process via shared-memory (pipes in Linux). In-memory communication is the fastest strategy for fuzzer and SUT to communicate, as it avoids the overhead of a real network stack, a simulated network stack, or shared memory.

As mentioned above, this approach only works with in-process fuzzing, which involves compiling the SUT code so that the fuzzer is directly embedded within it. LibFuzzer [2] uses this approach. In-process fuzzing not only reduces the overhead of communication over the network stack (O1), it also provides ways to tackle the overhead of context switches (O2) and the initialization and termination of the SUT (03), as discussed in Sect. 6.

## Mitigating context switching overhead (O2)

There are strategies to send input traces to the SUT that can affect the overheads associated with context switching. These strategies are

1. Sending input messages one by one.
2. Sending a sequence of messages in one go (reduces overhead O2, for every input trace).

### Sending input messages one by one

The classical approach of network protocol fuzzers (e.g., AFLNet, AFLNwe, StateAFL) sends input messages one by one to the SUT and receives the respective responses. However, this strategy adds overhead for context switching between the fuzzer and the SUT.

**Fig. 3:**
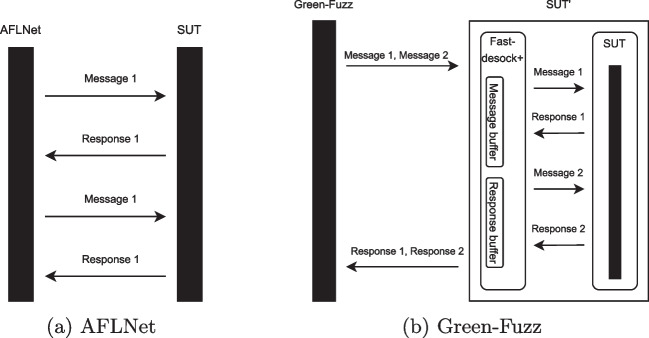
Sending a trace of input messages in AFLNet (**a**) vs Green-Fuzz (**b**). By sending all messages/entire trace in one go, unlike one by one in AFLNet, we save overhead from context switches and system-calls

### Sending input messages in one go (GreenFuzz)

In our previous article [31], we presented Green-Fuzz, a new strategy to reduce the context-switches between the fuzzer and SUT in the fuzzing process.

Current fuzzers for network protocols consider a trace of input messages $T=<m_1,m_2,...,m_i>$, and send the input message $m_i$ one by one to fuzz the SUT. By using the Green-Fuzz, we do not send input messages one by one but as a trace. We do this because when the fuzzer sends input messages one by one, the fuzzer has to call two (or more) system-calls for each input message and call the same number of system-calls to receive the respective response from the SUT. However, by sending the entire trace of input messages in one go, the number of system-calls is reduced: for a trace of input messages *T* with *n* messages, we only have the overhead once, instead of *n* times. This strategy can be applied to any network protocol fuzzer. However, because Green-Fuzz sends the whole trace in one go, there is a limitation where we assume that the fuzzer can decide on the input trace in advance. We applied this strategy on AFLNet [14].

To apply our strategy to AFLNet, we implemented another simulated socket library named *Fast-desock+*. Fast-desock+ intercepts and buffers the trace of input messages *T* sent by Green-Fuzz fuzzer and sends it to the SUT in one go. Likewise, it intercepts and saves all the SUT responses into a response buffer before forwarding them to the fuzzer.

The difference between Fast-desock+ and Desock+ is that Fast-desock+ also hooks *sendto*, *recvfrom*, and *setsockopt* to intercept and buffer trace of input messages and responses between the fuzzer and SUT.

Figure 3a shows the AFLNet interaction with the SUT, where the fuzzer sends each input message one by one. Figure 3b shows the Green-Fuzz interaction with the SUT, which sends a trace of the input messages to the SUT in one go.

## Mitigating SUT initialization and termination (O3)

When it comes to the overhead of initializing and termination the SUT (O3), we can distinguish four strategies:

1. Out-process fuzzing;
2. Snapshotting and forking (reduces overhead O3, for every input trace);
3. Persistent mode fuzzing (reduces overhead O3, for every input trace);
4. In-process fuzzing (reduces overhead O1 and O3, for every input message and trace).

### Out-process fuzzing

Here, the fuzzer and SUT run as separate processes. Most fuzzers, including most network protocol fuzzers, take this approach, for example, AFLNet [14] or StateAFL [22].

This approach’s advantage is that it does not require any modification of the SUT, unlike the persistent mode and in-process strategies below, which require manual work to change the SUT’s code. So, it is suited to situations where the source code is unavailable or SUT modification is complex.

Downsides are the overheads: in the communication between the two processes, i.e., O1 (using one of the techniques discussed in Sect. 4), in the context switches between the processes, i.e., O2, and the initialization and termination of the SUT process, i.e., O3.

### Snapshotting

Snapshotting consists of saving (or *snapshotting*) the SUT at a certain point to avoid the initialization and termination overheads.

For stateless systems, it is usually useful to snapshot the SUT after the initialization phase and just before handling the inputs to avoid unnecessary initialization and termination overhead.

For the stateful network SUT, snapshotting can do more. Fuzzers like Nyx-Net [1] use snapshots to capture the program state at a particular point and then avoid the costs of re-sending the entire trace to reach a certain state. Unfortunately, the snapshotting mechanisms themselves are often quite expensive.

### Persistent fuzzing

The basic idea behind persistent fuzzing is to avoid the overhead of initializing and terminating the SUT. To avoid these overheads, persistent fuzzing allows the SUT to process multiple inputs before shutting down and restarting.

This idea was initially applied only to fuzz stateless SUTs, like graphic routines that process a single JPEG and then terminate. Note that even if, for such SUTs, we might not have the overhead of a network connection (O1), we do have the overhead of initializing and terminating SUT processes (O3). Enabling the persistent mode for such systems requires the SUT modification. This is typically done by adding a while loop around the code that accepts and processes inputs. This allows the overhead O3 to be only once in a while (i.e., when the loop ends) and not for each input. Persistent fuzzing is known to give a huge speed-up. The AFL++ documentation claims that it is easily 10 or 20 times faster^4^—and hence the method of choice for the most fuzzing campaign.

Things are slightly different when we apply the idea of persistent fuzzing in network protocols. Network protocols already do *some* persistent fuzzing. They usually process several messages (a trace) without restarting the SUT. The persistent mode is still useful for stateful network SUT when we want to process several traces without restarting the SUT. We need a *soft* reset command to bring the SUT back to the initial state.

For example, AFL* [33] is a network protocol fuzzer based on AFL++ [23] persistent mode. By leveraging AFL++ persistent mode, AFL* avoids the overheads associated with initialization and termination. However, this strategy introduces the need to reset the SUT after sending each trace. Such resets can be *soft* or *hard*, depending on the nature of the SUT options. For example, a soft reset can be used if the SUT supports a *QUIT* command, eliminating reset-related overhead. A hard reset becomes mandatory for fuzzing stateful network SUT in scenarios lacking a soft reset, reintroducing significant overhead. This variance underscores the importance of understanding the specific requirements and capabilities of the protocol being fuzzed to optimize the efficiency of the AFL* strategy.

### In-process fuzzing

The idea behind in-process fuzzing is to run the fuzzer and the SUT into a single process. This reduces or even eliminates a lot of overheads: there are no context switches between SUT and fuzzer and communication can happen in-memory.^5^

In-process fuzzing typically goes hand in hand with persistent fuzzing, as it is ideal not to start a new process for each input (in the case of stateless SUT) or each trace (in the case of stateful network SUT). A critical distinction between persistent mode and in-process fuzzing lies in their integration with the SUT. In-process fuzzing allows the fuzzing and the SUT to operate as a single process, avoiding context switches.

In-process fuzzing [2, 8] (like persistent mode) requires the users to modify the SUT code manually. After the modification, the SUT can send multiple messages, avoiding the overhead of initialization and termination. This results in a significantly faster fuzzing process. While in-process fuzzing often uses in-memory communication to achieve efficiency, it is not always the case. However, it is important to note that employing an in-memory fuzzing strategy depends on using an in-process strategy, highlighting the intertwined nature of these strategies for enhancing fuzzing effectiveness.

**Table 2:** Comparing strategies presented in Sects. 4, 5, and 6 and their performance gain on LightFTP protocol

Strategy	Reduction in O1		Reduction in O2		Reduction in O3		Impact
Simulated network stack	50%	15 ms	–	–	–	–	+
Shared-memory	70%	21 ms	–	–	–	–	++
All-in-one-go	–	–	78%	8 ms	–	–	++
Persistent mode	–	–	–	–	96%	45 ms	+++
In-memory	92%	28 ms	100%	10 ms	96%	45 ms	++++

The performance gain percentage is per overhead root cause. For example, simulated network stack reduces the network stack overhead by 50%. We also show the absolute gained time is in milliseconds. The dash (-) means no performance gain

## Analyzing and comparing strategies

This section analyzes the effectiveness of each strategy presented in Sects. 4, 5, and 6. For each strategy, we measure the overheads of the individual system-calls, context-switching, and time spent on the process. Moreover, we compare these results with those presented in our previous paper about Desock+ and Green-Fuzz.

### Evaluation criteria

We evaluate and compare the strategies discussed before to measure each strategy’s potential overhead reduction. In our analysis, we

- Considered the time taken by network-related system calls from fuzzer and SUT;
- Considered the context switching between the SUT and compared the time taken for Green-Fuzz with its baseline;
- Added break points after initialization and termination to see how long it takes for the SUT to process the input messages;
- Monitored the system-calls and execution time for both in-process and out-process fuzzers.

Table 2 presents the reduction in overhead for processing a single input trace for each specific overhead root cause. We expect the performance gain to grow along with the number of traces taken into account. In front of each performance gain percentage, we have also shown the time gained by each strategy. We have also shown the impact of each strategy on the performance gain (more + means more performance gain), which relates to the percentage of the performance gain for each overhead root cause. According to Table 2, the in-memory and in-process strategy demonstrates the highest overhead reduction among all the strategies evaluated. However, as outlined in Sect. 4, this significant performance gain requires modifications to the SUT, which might not always be feasible.

### Evaluation of simulated network stack (Desock+)

We used AFLNet with and without Desock+ to evaluate the technique’s effectiveness. Both sets of fuzzing experiments have been done with an identical setup on the five SUTs from ProFuzzBench [13], a benchmark framework for stateful systems.

We ran our experiment five times for an hour. Table 3 shows that using Desock+ increases the fuzzing speed up to four times.

**Table 3:** Speed in message per second, of AFLNet with and without Desock+ on ProFuzzBench [13]

SUT	AFLNet	AFLNet with Desock+	Speed up
lightFTP	12	49	$+308\%$
dnsmasq	15	19	$+26\%$
live555	14	29	$+107\%$
dcmqrscp	17	21	$+23\%$
tinydtls	12	19	$+58\%$

### Evaluation of sending multiple messages in one go (GreenFuzz)

Table 4 shows the execution speed of Green-Fuzz and AFLNet using Desock+. Five of the ten SUTs included in ProFuzzBench use the socket options our fuzzer supports. We fuzzed the SUTs for an hour and repeated our experiment to ensure reliable numbers. The results show that the trace of input messages fuzzed per second is higher when using Green-Fuzz than AFLNet using Desock+.

We used *ptrace* to monitor system-calls that source the overhead while fuzzing. Table 5 shows the absolute overhead difference, where we can see Green-Fuzz decreases overhead in *recvfrom*, *sendto*, *setsockopt*, and *connect* system-calls. There is no change in overhead regarding the *kill* and *clone* system-calls because both AFLNet and Green-Fuzz are out-process fuzzers and have to use these system-calls for each trace of input messages.

**Table 4:** Speed in message per second of AFLNet with Desock+ and Green-Fuzz on ProFuzzBench

SUT	AFLNet with Desock+	Green-Fuzz	Speed up
lightFTP	49	64	$+30\%$
dnsmasq	19	19	$0\%$
live555	29	31	$+6\%$
dcmqrscp	21	25	$+19\%$
tinyDTLS	19	34	$+78\%$

## Related work

Using grey-box fuzzing solutions to test network services has become a popular research topic. One example is Peach* [19], which combines code coverage feedback with the original Peach [20] fuzzer to test Industrial Control Systems (ICS). It collects code coverage information during fuzzing and uses Peach’s capabilities to generate more effective test cases.

IoTHunter [21] applies grey-box fuzzing for network services in IoT devices. It uses code coverage to guide the fuzzing process and implements a multi-stage testing approach based on protocol state feedback.

AFLNet [14] is a grey-box fuzzer for protocol implementations that uses state feedback to guide fuzzing. It acts as a client which mutates and forwards messages to the SUT.

StateAFL [22] is a variation of AFLnet that uses a memory state to represent the service state. It instruments the target server during compilation and determines the current protocol state at runtime. It gradually builds a protocol state machine to guide the fuzzing process.

**Table 5:** Comparison of absolute system-call overhead between AFLNet and Green-Fuzz on LightFTP protocol

System-call	AFLNet	Green-Fuzz	Overhead difference
*clone*	$n \times 6.5$	$n \times 6.5$	$0\%$
*kill*	$n \times 8.7$	$n \times 8.7$	$0\%$
*recvfrom*	$n \times m \times 1.2$	$n \times 1.2$	$-80\%$
*sendto*	$n \times m \times 1.3$	$n \times 1.3$	$-80\%$
*setsockopt*	$n \times m \times 0.1$	$n \times 0.1$	$-80\%$
*connect*	$n \times 11$	$n \times 4$	$-63\%$

The times are in milliseconds (from an example SUT) and shown in the format of $n \times m \times time$ where *n* is the number of traces and *m* is the number of messages in one trace, which is 5 in this experiment

### Related work with Desock+

Zeng et al. [18] also made a simulated socket library, named Desockmulti, to avoid network communication overhead when fuzzing network protocols. However, compared to Desock+, Desockmulti does not support *connect* and *accept4* system-calls, which limits its applicability.

Maier et al. [12] introduced the Fuzzer in the Middle (FitM) for fuzzing network protocols. Instead of using a simulated socket library, FitM intercepts the emulated system-calls inside the QEMU emulator and sends the input messages to the SUT without the network communication overhead. Since FitM has emulation overhead, it is slower than our approach but can fuzz both the client and server of a network protocol as the SUT.

There are also ad-hoc approaches [5, 6, 26] that manually modify the SUT to get rid of the network stack.

### Related work with Green-Fuzz

Nyx-Net [15] uses hypervisor-based snapshot fuzzing incorporated with the emulation of network functionality to handle network traffic. Nyx-Net uses a customized kernel module, a modified version of QEMU and KVM, and a custom VM configuration. Nyx-Net also contains a custom networking layer miming certain POSIX network functionalities, which currently needs more support for complicated network targets. In contrast, Green-Fuzz adopts a user-mode approach that avoids complexity. Green-Fuzz is also an orthogonal approach to be added to Nyx-Net to speed up the fuzzing speed.

As explained in Sect. 6, in-process fuzzing [2, 8] allows not to restart or fork the SUT for each trace or message and to mutate the messages in-memory, avoiding the network communication overhead. Although this method performs better than our approach (around 200 to 300 times in our experiments), it involves manual work to specify the exact position of variables inside the memory. Another issue of in-process fuzzing is that it usually can not test the whole system because of the fuzzing loop that is defined for the harness.

## Limitations and future work

Currently, Desock+ only works with the SUTs using system-calls and their arguments shown in Table 1. Some SUTs use other socket options. For example, input arguments for *epoll* system-call must be simulated in Desock+ to work correctly if the SUT is using this specific system-call. Since part of Green-Fuzz is based on Fast-desock+, these limitations apply to Green-Fuzz. We plan to improve Green-Fuzz to fuzz network protocols such as OPC-UA [24] and Modbus [25] protocols. To fuzz protocols that require a handshake, the Green-Fuzz needs a minor modification to do the handshake before sending the whole trace in one go.

In this article, we applied Desock+ and Green-Fuzz to AFLNet. However, these general solutions can be applied to any fuzzer that uses network sockets and does not need feedback after every single message, as [11] does. Good candidates for future integration are SGPFuzzer [17] and Nyx-net [15].

## Recommendations for software developers

To reduce the overhead and complexity of the SUT, developers might consider to

- Incorporating a restart message: this feature would allow the fuzzer to send a specific command to reset the state of the protocol and refresh all variables. It is beneficial in stateful fuzzing, as it permits quick resetting without requiring complete process restart;
- Disabling encryption mechanisms: this would help fuzzers fuzz cryptographic protocols without the need to deal with encrypted messages, keys, or cryptographic checks;
- Supporting standard I/O communication: enabling standard inputs/outputs communication channels (along with the network stack one) will allow faster and more direct data transmission between the fuzzer and the SUT.

## Conclusions

In conclusion, fuzzing emerges as a powerful method for uncovering bugs and security flaws within software systems. Yet, its application to network protocols has faced limitations, primarily due to reduced throughput. This article delves into the root causes of overhead in fuzzing network protocols, thoroughly examining strategies to reduce or avoid these strategies. We explored and categorized strategies, assessing the advantages and disadvantages of each to offer a comprehensive view. Our analysis, including insights from our prior article and additional research, indicates that in-memory and in-process fuzzing strategies are the fastest fuzzing strategies. However, this efficiency often requires modifications to the SUT, which may not always be desirable or feasible. For scenarios where modifying the SUT is not an option, employing an out-process fuzzer, particularly one that utilizes sending a whole trace in one go and using shared memory, presents the next best strategy for enhancing fuzzing speed. Our overview provides better insight into choosing the appropriate strategies for fuzzing network protocols. This paves the way for more effective and efficient identification of vulnerabilities in network protocols.

## Data Availability

All the data and materials are available at https://github.com/behnamandarz/GreenFuzz

## Footnotes

1 Continuous Integration/Continuous Deployment.

2 This is an estimation based on our experience with out-process fuzzing using AFL fuzzer.

3 A loopback address is a unique IP address that is used to refer to the localhost.

4 https://github.com/AFLplusplus/AFLplusplus/blob/sable/instrumentation/README.persistent_mode.md

5 Be aware that in-process and in-memory are not synonyms.
